# Chronic cigarette smoking is associated with increased arterial stiffness in men and women: evidence from a large population-based cohort

**DOI:** 10.1007/s00392-022-02092-1

**Published:** 2022-09-06

**Authors:** Omar Hahad, Volker H. Schmitt, Natalie Arnold, Karsten Keller, Jürgen H. Prochaska, Philipp S. Wild, Andreas Schulz, Karl J. Lackner, Norbert Pfeiffer, Irene Schmidtmann, Matthias Michal, Jörn M. Schattenberg, Oliver Tüscher, Andreas Daiber, Thomas Münzel

**Affiliations:** 1 Department of Cardiology-Cardiology I, University Medical Center of the Johannes Gutenberg-University Mainz, Langenbeckstraße 1, 55131 Mainz, Germany; 2 German Center for Cardiovascular Research (DZHK), Partner Site Rhine-Main, Mainz, Germany; 3 Department of Cardiology, German Center for Cardiovascular Research (DZHK), partner site Hamburg/Kiel/Luebeck, Hamburg, Germany; 4 Center for Thrombosis and Hemostasis, University Medical Center of the Johannes Gutenberg-University Mainz, Mainz, Germany; 5 Medical Clinic VII, Department of Sports Medicine, University Hospital Heidelberg, Heidelberg, Germany; 6 Preventive Cardiology and Preventive Medicine, Department of Cardiology, University Medical Center of the Johannes Gutenberg-University Mainz, Mainz, Germany; 7 Institute of Clinical Chemistry and Laboratory Medicine, University Medical Center of the Johannes Gutenberg-University Mainz, Mainz, Germany; 8 Department of Ophthalmology, University Medical Center of the Johannes Gutenberg-University Mainz, Mainz, Germany; 9 Institute of Medical Biostatistics, Epidemiology and Informatics, University Medical Center of the Johannes Gutenberg-University Mainz, Mainz, Germany; 10 Department of Psychosomatic Medicine and Psychotherapy, University Medical Center of the Johannes Gutenberg-University Mainz, Mainz, Germany; 11 Metabolic Liver Research Program, I. Department of Medicine, University Medical Center of the Johannes Gutenberg-University Mainz, Mainz, Germany; 12 Department of Psychiatry and Psychotherapy, University Medical Center of the Johannes Gutenberg-University Mainz, Mainz, Germany

**Keywords:** Cigarette smoking, Arterial stiffness, Wave reflection, Stiffness index, Augmentation index, Sex-specific, Population-based study

## Abstract

**Background:**

Cigarette smoking is a threat to global human health and a leading cause of the cardiovascular disease (CVD) morbidity and mortality. Importantly, sex-specific differences in smoking-induced arterial stiffness, an early key event in the development of atherosclerotic CVD, remain still elusive. Thus, this study sought out to investigate sex-specific associations between smoking and measures of arterial stiffness.

**Methods and results:**

Overall, 15,010 participants (7584 men and 7426 women aged 35–74 years) of the Gutenberg Health Study were examined at baseline during 2007–2012. Smoking status, pack-years of smoking, and years since quitting smoking were assessed by a standardized computer-assisted interview. Arterial stiffness and wave reflection were determined by stiffness index (SI) and augmentation index (AI). In the total sample, 45.8% had never smoked, 34.7% were former smokers, and 19.4% were current smokers. Median cumulative smoking exposure was 22.0 pack-years in current male smokers and 16.0 in current female smokers. In general, multivariable linear regression models adjusted for a comprehensive set of confounders revealed that smoking status, pack-years of smoking, and years since quitting smoking were dose-dependently associated with markers of arterial stiffness. In sex-specific analyses, these associations were overall more pronounced in men and SI was stronger related to the male sex, whereas differences between men and women in the case of AI appeared to be less substantial.

**Discussion:**

The present results indicate that chronic smoking is strongly and dose-dependently associated with increased arterial stiffness in a large population-based cohort regardless of sex but with a stronger association in men.

**Supplementary Information:**

The online version contains supplementary material available at 10.1007/s00392-022-02092-1.

## Introduction

Cigarette smoking represents one of the biggest (preventable) threats to public health. It is a leading cause of morbidity, disability, and death worldwide. Approximately every six seconds one person dies due to tobacco and sequelae, accounting for one of five deaths worldwide [1]. In line, a recent report from the World Health Organization (WHO) concluded that tobacco use kills up to half of its users and worldwide more than 8 million people each year, a major part caused by cardiovascular diseases (CVD) [2]. Among the risk factors for CVD, exposure to cigarette smoke is presumably one of the most complex and sparsely understood risk factors, containing more than 4,000 identified chemical compounds with a wide range of toxicity and sizes from atoms to particulate matter [3]. In particular, sex-specific differences in smoking-induced pathophysiological mechanisms remain largely unknown. There is a large body of evidence suggesting that cigarette smoking disturbs vascular endothelial homeostasis by reducing the bioavailability of vascular nitric oxide (NO), mainly via the formation of oxidative stress and inflammatory processes [4]. Since the formation of NO plays a key role for the endothelium to perpetuate its vasodilatory, antithrombotic, anti-inflammatory, and antioxidant functions, this pathological state promotes endothelial dysfunction and atherosclerotic plaque formation, ultimately leading to structural changes in the arterial wall and arterial stiffening [5, 6]. Importantly, arterial stiffness has been shown to be an independent risk factor for CVD and all-cause mortality after adjustment for traditional cardiovascular risk factors [7], in addition to being a more potent CVD risk factor in women than in men [8]. Moreover, the assessment of aortic stiffness in the Framingham Heart Study improved risk prediction for the first cardiovascular event when added to a standard risk factor model [9]. Of note, smoking exposure even at low intensity in teenage years was individually and in combination with alcohol consumption associated with increased arterial stiffness [10, 11]. Notable, current smoking does not only influence the arterial vascular system, but also the venous system with an increased risk for venous thromboembolism [12]. However, substantial sex differences were observed in the development of arterial stiffness, not only in the time course of aging-related arterial stiffness and the associated risk of CVD, but also in the context of CVD risk factors such as smoking, diabetes mellitus, and obesity [13]. Thus, the aim of the present study was to examine the sex-specific associations between smoking and markers of arterial stiffness and wave reflection in a large population-based cohort.

## Methods

### Study procedure and sample

As described by Wild et al. in detail, the Gutenberg Health Study (GHS) is a population-based, prospective, observational single-center cohort study from Mid-Western Germany, including residents of the City of Mainz and the region Mainz-Bingen [14]. At baseline, 15,010 individuals, aged 35 to 74 years and stratified 1:1 for sex, residence (urban and rural), and decades of age, were examined between April 2007 and April 2012 at the University Medical Center Mainz, Germany. All procedures in the GHS were approved by the ethics committee of the Statutory Physician Board of the State Rhineland-Palatinate, Germany (reference number 837.020.07(5555)) and the local data safety commissioners. The study design was in line with the Declaration of Helsinki and principles outlined in the recommendations for Good Clinical and Epidemiological Practice. Participants were included after written informed consent. The GHS mainly focuses on the analysis of cardiovascular risk factors and improvement of risk stratification. Moreover, determinants of metabolic, ophthalmological, cancer, immune system, and mental diseases are of interest. Participants underwent a range of standardized examinations according to standard operating procedures, including a comprehensive assessment of clinical, laboratory, lifestyle, psychosocial, and environmental parameters. Quality control of all data and screening for completeness according to predefined algorithms and plausibility criteria were performed by a central data management unit.

### Smoking variables

A standardized computer-assisted interview was conducted to collect information about smoking history, including smoking status, pack-years of smoking in former and current smokers, and years since quitting in former smokers.

With respect to their smoking status, participants were categorized as non-smokers, former smokers, or current smokers. Current smoking comprised regular or daily smoking (at least 1 cigarette per day, 7 per week, or 1 pack per month) for at least the past 6 months. The group of non-smokers included non-daily and non-regular smokers. Former smokers were those who had a history of smoking (regular or daily smoking) for longer than 6 months and who were no current smokers. To determine the pack-years of smoking, current and former smokers were asked for the year of smoking initiation/cessation and average consumption of cigarettes per day. This was calculated as the average number of cigarettes smoked per day divided by 20 (a pack) and multiplied by the number of years smoked. Pack-years of smoking as a cumulative exposure indicator of smoking burden were used to adequately address both intensity and duration of smoking. Exposure to passive smoke in never and former smokers was defined as being exposed to cigarette smoke at home, workplace, and/or elsewhere (e.g. bars, clubs, restaurants) for at least half an hour per day. Participants were asked not to smoke for at least 8 h prior to the examination to avoid the acute effects of smoking.

### Assessment of arterial stiffness and wave reflection

Briefly, stiffness index (SI) and augmentation index (AI) were assessed in dark, air-conditioned rooms (room temperature 23 to 25 °C) after at least 5 min of rest in the supine position. All participants were advised to come in a fasted state of at least 8 h. Subjects were particularly advised to refrain from nicotine, caffeine, alcohol, vitamins, and physical activity before measurement. The AI was determined by digital volume plethysmography using an EndoPat 2000 device (Itamar Medical, Caesarea, Israel), which is based on the fingertip measurement of pulsatile volume changes. The AI is automatically calculated by identifying the early (P1) and late systolic peak (P2) with the following formula: (P2 − P1)/P1 × 100. The SI was assessed by digital photoplethysmography (PCA2 device; Carefusion) and used as a measure for systemic arterial stiffness. Briefly, a volume pulse waveform with an early systolic and a second diastolic/reflected peak was recorded by the transmission of infrared light through the finger pulp. The SI was calculated as the subject’s height (meters) divided by the time difference between these 2 peaks (so‐called “peak‐to‐peak time”) in seconds. Both measurements were performed simultaneously according to standard operating procedures with calibration for the device used and detailed quality control. Detailed description of this method has been provided recently [15, 16].

### Definitions of further included variables

Prevalent CVD were assessed on basis of medical history or diagnosed during the study visit. Prevalent CVD was defined as the presence of any of the following diseases: coronary artery disease, peripheral artery disease, myocardial infarction, congestive heart failure, stroke, or atrial fibrillation. Medication use was assessed on basis of medical records as well as personal reports and was categorized according to the Anatomical Therapeutic Chemical Classification System [17]. Arterial hypertension was diagnosed by the intake of antihypertensive drugs or a mean systolic blood pressure ≥ 140 mmHg or diastolic blood pressure ≥ 90 mmHg at rest (average of 2^nd^ and 3^rd^ standardized measurement after 8 and 11 min of rest). Waist-to-height ratio was calculated as waist circumference in centimeters divided by the body height in centimeters. Heart rate was assessed by the mean of the 2nd and 3rd measurements at rest. Diabetes mellitus was defined by any of the following: diagnosed by a physician, antidiabetic treatment, fasting blood glucose level (overnight fast of at least 8 h) ≥ 126 mg/dL, non-fasting blood glucose level (less than 8 h of fasting) ≥ 200 mg/dL, or HbA_1c_ ≥ 6.5%. Dyslipidemia was present in the case of a physician diagnosis of dyslipidemia, low-density lipoprotein cholesterol/high-density lipoprotein cholesterol ratio > 3.5, or triglycerides ≥ 150 mg/dL. A positive history of myocardial infarction or stroke was recorded in a female first-degree relative ≤ 65 years or in a male first-degree relative ≤ 60 years. Socioeconomic status was assessed in accordance with Lampert et al. by a validated index score (ranging from 3 to 21), providing information about educational background, current occupation, and salary [18]. The categories “beneath” and “above tolerable limit” were used to characterize alcohol consumption (cut-offs were > 24 g per day for men and > 12 g per day for women) [19]. A Patient Health Questionnaire (PHQ-9) score ≥ 10 was used for the caseness of depression [20]. The Short Questionnaire to Assess Health-enhancing physical activity (SQUASH), which comprises questions on multiple activities to calculate an index score (total minutes of activity multiplied by intensity), was used to determine physical activity [21]. Postmenopausal status, intake of oral contraceptives, and hormone replacement therapy in women were assessed by medical records and personal reports.

### Statistical analysis

The main analyses were carried out sex-specifically, while the supplemental material provides analyses for the total sample. Study sample characteristics are shown according to sex as absolute and relative frequency for categorical variables and as mean value and standard deviation or median with 25th and 75th percentiles for continuous variables. Linear regression analysis with corresponding beta estimates (*β*) was used to assess the association between smoking status, pack-years of smoking in former and current smokers, and years since quitting smoking in former smokers with markers of arterial stiffness and wave reflection. Models were adjusted sequentially:

Model 1 (basic model): Adjustment for age (continuous). Linear regressions for the parameter AI were additionally adjusted for height and heart rate (both as continuous variables). In women, further adjustment for postmenopausal status, intake of oral contraceptives, and hormone replacement therapy were done (all binary).

Model 2 (comprehensive model): Further adjustment for arterial hypertension (binary), diabetes mellitus (binary), waist-to-height ratio (continuous), dyslipidemia (binary), family history of myocardial infarction or stroke (binary), socioeconomic status (binary), alcohol consumption above the tolerable limit (binary), depression (binary), physical activity (continuous), passive smoking (binary, when applicable), smoking prior to the examination (binary, when applicable), prevalent CVD (binary, as defined), and medication use (binary for diabetic drugs, antithrombotic agents, antihypertensive drugs, diuretics, beta-blockers, calcium channel blockers, agents acting on the renin–angiotensin–aldosterone system, and lipid modifying agents).

All regression analyses were performed on subjects with complete data for smoking exposure variables and other confounding variables included in the models. To confirm the robustness of the cross-sectional results, we further prospectively (follow-up data obtained from 2012 to 2017) analyzed the association between smoking status and markers of arterial stiffness and wave reflection. Effect estimates are given with 95% confidence intervals (CI) with corresponding *p* values. Because of the explorative nature of the study, *p* values should be treated as a continuous measure of the statistical strength of an association, and they are therefore reported exactly. The statistical data analyses were performed using the software R, version 4.1.0 (http://www.r-project.org/).

## Results

### Study sample characteristics

In total, 6863 (45.8%) individuals were never-smokers, while 5201 (34.7%) smoked in former times, and 2911 (19.4%) were current smokers (Supplementary Table S1). As evident in Table 1, men had in general a higher socioeconomic status, lower prevalence of depression, and higher prevalence of alcohol consumption above the tolerable limit compared to women. Men were more likely to be current smokers and had higher cumulative smoking exposure in terms of pack-years. Concerning traditional cardiovascular risk factors and CVD, women expressed an overall favorable risk profile with lower prevalences of arterial hypertension, diabetes mellitus, dyslipidemia, congestive heart failure, coronary artery disease, myocardial infarction, stroke, and peripheral artery disease. Consequently, overall use of medication was higher among men than in women. With regard to markers of arterial stiffness, men had higher SI, whereas women displayed higher AI. There was a steady increase of SI and AI from never-smokers to former and current smokers among men (SI: 8.01 ± 2.28 m/s–8.54 ± 2.34 m/s–8.64 ± 2.31 m/s; AI: 7.50 ± 17.46%–13.40 ± 18.25%–14.73 ± 19.54%) and women (SI: 6.61 ± 1.66 m/s–6.69 ± 1.66 m/s–6.79 ± 1.80 m/s; AI: 22.32 ± 19.97%–23.95 ± 20.01%–27.46 ± 22.74%).

**Table 1:** Characteristics of the study sample stratified by sex (*N* = 15,010)

	Men (*N* = 7584)	Women (*N* = 7426)	All (*N* = 15,010)
Characteristic			
Age—years	55.3 ± 11.1	54.8 ± 11.1	55.0 ± 11.1
Physical activity**	7.38 ± 4.32	7.35 ± 3.61	7.37 ± 4.00
Heart rate—beats per minute	68.0 ± 11.1	70.0 ± 10.5	69.0 ± 10.8
Height—cm	177 ± 7	164 ± 7	170 ± 10
Waist-to-height ratio^#^	0.56 ± 0.07	0.55 ± 0.09	0.56 ± 0.08
Postmenopausal status—no. (%)	–	5,015 (67.7)	5,015 (33.4)
Hormone replacement therapy—no. (%)	–	806 (10.9)	806 (5.4)
Intake of oral contraceptives—no. (%)	–	448 (6.1)	448 (3.0)
Alcohol consumption above tolerable limit—no. (%)^‡^	1888 (25.0)	1476 (19.9)	3364 (22.5)
Socioeconomic status^†^	13.59 ± 4.62	12.16 ± 4.21	12.89 ± 4.48
Depression—no. (%)§	450 (6.1)	683 (9.4)	1,133 (7.7)
Smoking			
Current smoking	1,576 (20.8)	1,335 (18.0)	2,911 (19.4)
Pack-years (among current smokers)	22.0 (9.90/36.0)	16.0 (7.20/26.2)	18.7 (8.40/31.8)
Years since quitting (among former smokers)	18.0 (8.00/30.0)	17.0 (7.00/28.0)	18.0 (8.00/29.0)
Passive smoking—no. (%) (among never and former smokers)	994 (13.1)	892 (12.0)	1886 (12.6)
Smoked < 12 h prior to examination—no. (%)	1145 (15.1)	1001 (13.5)	2146 (14.3)
Traditional cardiovascular risk factors—no. (%)			
Arterial hypertension	4142 (54.6)	3324 (44.8)	7466 (49.8)
Diabetes mellitus	863 (11.4)	532 (7.2)	1395 (9.3)
Dyslipidemia	3257 (43.1)	1919 (25.9)	5176 (34.6)
Family history of myocardial infarction or stroke	1532 (20.2)	1789 (24.1)	3321 (22.1)
Cardiovascular comorbidities—no. (%)			
Congestive heart failure	728 (9.6)	424 (5.7)	1,152 (7.7)
Coronary artery disease	484 (6.5)	157 (2.1)	641 (4.3)
Myocardial infarction	339 (4.5)	103 (1.4)	442 (3.0)
Stroke	182 (2.4)	97 (1.3)	279 (1.9)
Atrial fibrillation	1,025 (13.5)	1,679 (22.6)	2,704 (18.0)
Peripheral artery disease	277 (3.7)	227 (3.1)	504 (3.3)
Measurements of arterial stiffness			
Stiffness index—m/s	8.35 ± 2.32	6.66 ± 1.69	7.54 ± 2.21
Augmentation index—%	11.39 ± 18.48	23.73 ± 20.60	17.41 ± 20.49
Medication—no. (%)^§§^			
Diabetic drugs (A10)	594 (7.9)	331 (4.5)	925 (6.2)
Antithrombotic agents (B01)	1,191 (15.9)	658 (8.9)	1,849 (12.4)
Antihypertensive drugs (C02)	83 (1.1)	72 (1.0)	155 (1.0)
Diuretics (C03)	393 (5.2)	397 (5.4)	790 (5.3)
Beta-blockers (C07)	1,313 (17.5)	1,224 (16.6)	2,537 (17.1)
Calcium channel blocker (C08)	619 (8.3)	470 (6.4)	1,089 (7.3)
Agents acting on the renin–angiotensin–aldosterone system (C09)	2,054 (27.4)	1,489 (20.2)	3,543 (23.8)
Lipid modifying agents (C10)	1,175 (15.7)	809 (11.0)	1,984 (13.4)

Plus-minus values are means ± standard deviation and two values in parentheses are medians with 25th and 75th percentiles

**Physical activity score was calculated by multiplying total minutes of activity by the intensity score displayed per 1000 units with higher values indicating higher physical activity

^#^Waist-to-height ratio is the waist circumference divided by the body height in centimeters

^‡^Alcohol consumption above tolerable limit denotes > 24 g per day for men and > 12 g per day for women

^†^Socioeconomic status score ranges from 3 to 21 with higher values indicating higher status

^§^Caseness of depression was indicated by a PHQ-9 score ≥ 10

^§§^Medication is labelled with the anatomical therapeutic chemical code

### Association between measures of arterial stiffness and smoking exposure

In the total sample, smoking status was independently associated with SI and AI in a dose-dependent manner after comprehensive adjustment (Supplementary Table S2 and Fig. S1). Sex-specific analyses showed in general a comparable pattern with higher effect estimates for men compared to women, while smoking status was not predictive of SI in model 2 after comprehensive adjustment (Table 2). The prospective analyses on the association between smoking status and SI and AI could confirm the cross-sectional results (Table 3).

**Table 2:** Sex-specific associations between smoking status and markers of arterial stiffness

	Model 1** Beta estimate [95% CI]	*P* value	Model 2^#^ Beta estimate [95% CI]	*P* value
Estimates for stiffness index				
Men				
Never smoking (ref.)	–	–	–	–
Current smoking	0.83 [0.69; 0.98]	** < 0.0001**	0.55 [0.29; 0.80]	** < 0.0001**
Former smoking	0.29 [0.17; 0.41]	** < 0.0001**	0.26 [0.12; 0.39]	**0.00024**
Women				
Never smoking (ref.)	–	–	–	–
Current smoking	0.43 [0.31; 0.54]	** < 0.0001**	0.15 [− 0.063; 0.37]	0.16
Former smoking	0.095 [0.0016; 0.19]	**0.046**	0.073 [− 0.035; 0.18]	0.18
Estimates for augmentation index				
Men				
Never smoking (ref.)	–	–	–	–
Current smoking	9.6 [8.5; 11]	** < 0.0001**	6.9 [5.1; 8.7]	** < 0.0001**
Former smoking	3.3 [2.4; 4.1]	** < 0.0001**	2.8 [1.8; 3.8]	** < 0.0001**
Women				
Never smoking (ref.)	-	-	-	-
Current smoking	7.2 [5.9; 8.5]	** < 0.0001**	4.9 [2.5; 7.4]	** < 0.0001**
Former smoking	1.6 [0.47; 2.7]	**0.0051**	2.4 [1.1; 3.6]	**0.00022**

Beta estimates and 95% confidence intervals are derived from a linear regression model modelling for arterial stiffness. Current and former smoking were compared to never smoking (reference category)

Statistically significant *P* values (*P* < 0.05) are given in bold

**Model 1 was adjusted for age and augmentation index was additionally adjusted for height and heart rate. In women further adjustment for postmenopausal status, intake of oral contraceptives, and hormone replacement therapy was done

^#^Model 2 was additionally adjusted for arterial hypertension, waist-to-height ratio, diabetes mellitus, dyslipidemia, family history of myocardial infarction or stroke, socioeconomic status, alcohol consumption, physical activity, depression, passive smoking, smoking prior to the examination, prevalent cardiovascular disease (compromising congestive heart failure, coronary artery disease, myocardial infarction, stroke, atrial fibrillation, and peripheral artery disease), and medication use (diabetic drugs, antithrombotic agents, antihypertensives, diuretics, beta-blockers, calcium channel blocker, agents acting on the renin–angiotensin–aldosterone system, and lipid modifying agents)

**Table 3:** Prospective associations between smoking status and markers of arterial stiffness

	Model 1** Beta estimate [95% CI]	*p* value	Model 2# Beta estimate [95% CI]	*p* value
Estimates for stiffness index				
All				
Never smoking (ref.)	–	–	–	–
Current smoking	0.45 [0.26; 0.65]	** < 0.0001**	0.42 [0.20; 0.65]	**0.00023**
Former smoking	0.19 [0.035; 0.34]	**0.016**	0.18 [− 0.0050; 0.36]	0.057
Men				
Never smoking (ref.)	–	–	–	–
Current smoking	0.54 [0.26; 0.82]	**0.00017**	0.61 [0.28; 0.93]	**0.00027**
Former smoking	0.24 [0.022; 0.47]	**0.032**	0.29 [0.032; 0.55]	**0.028**
Women				
Never smoking (ref.)	–	–	–	–
Current smoking	0.31 [0.045; 0.57]	**0.021**	0.18 [− 0.12; 0.48]	0.24
Former smoking	0.13 [− 0.085; 0.34]	0.24	0.059 [− 0.19; 0.31]	0.65
Estimates for augmentation index				
All				
Never smoking (ref.)	–	–	–	–
Current smoking	2.7 [1.5; 4.0]	** < 0.0001**	3.0 [1.6; 4.5]	** < 0.0001**
Former smoking	− 0.092 [− 1.1; 0.91]	0.86	− 0.30 [− 1.4; 0.85]	0.61
Men				
Never smoking (ref.)	–	–	–	–
Current smoking	2.4 [0.78; 3.9]	**0.0035**	2.9 [1.1; 4.7]	**0.0020**
Former smoking	− 0.72 [− 2.0; 0.52]	0.25	− 1.1 [− 2.6; 0.34]	0.13
Women				
Never smoking (ref.)	–	–	–	–
Current smoking	3.0 [0.99; 5.1]	**0.0038**	3.2 [0.93; 5.5]	**0.0059**
Former smoking	0.43 [− 1.2; 2.1]	0.61	0.34 [− 1.5; 2.2]	0.72

Beta estimates and 95% confidence intervals are derived from a linear regression model modelling for arterial stiffness. Current and former smoking were compared to never smoking (reference category)

Statistically significant *P* values (*P* < 0.05) are given in bold

**Model 1 was adjusted for baseline stiffness index/augmentation index and age (and sex in the analyses for all). Augmentation index was additionally adjusted for height and heart rate. In women further adjustment for postmenopausal status, intake of oral contraceptives, and hormone replacement therapy was done

^#^Model 2 was additionally adjusted for arterial hypertension, waist-to-height ratio, diabetes mellitus, dyslipidemia, family history of myocardial infarction or stroke, socioeconomic status, alcohol consumption, physical activity, depression, passive smoking, prevalent cardiovascular disease (compromising congestive heart failure, coronary artery disease, myocardial infarction, stroke, atrial fibrillation, and peripheral artery disease), and medication use (diabetic drugs, antithrombotic agents, antihypertensives, diuretics, beta-blockers, calcium channel blocker, agents acting on the renin–angiotensin–aldosterone system, and lipid modifying agents)

Likewise, increasing number of pack-years in the current (Supplementary Table S3 and Fig. S2) as well as in former smokers (Supplementary Table S6) was independently associated with SI and AI in a dose-dependent manner in the total sample. The pattern of results found for the impact of heavy smoking in current smokers was similar, showing that effect estimates for SI and AI were more than two-fold increased when comparing < 20 vs. ≥ 20 pack-years (Supplementary Table S4). Sex-specific analyses revealed pack-years of smoking in the current (Table 4) and former smokers (Table 5) as well as heavy smoking (Table 6) to be independently associated with SI and AI in a dose-dependent manner with lower and overall less monotone effect estimates for women than men.

**Table 4:** Sex-specific associations between pack-years of smoking in current smokers and markers of arterial stiffness

	Model 1** Beta estimate [95% CI]	*P* value	Model 2# Beta estimate [95% CI]	*P* value
Pack-years of smoking in current smokers	Estimates for stiffness index			
Men				
Never smoking (ref.)	–	–	–	–
> 0– < 10	0.41 [0.16; 0.67]	**0.0015**	0.32 [0.017; 0.62]	**0.038**
≥ 10– < 20	0.78 [0.50; 1.1]	** < 0.0001**	0.89 [0.48; 1.3]	** < 0.0001**
≥ 20– < 30	0.94 [0.64; 1.2]	** < 0.0001**	1.1 [0.71; 1.6]	** < 0.0001**
≥ 30	1.2 [0.95; 1.4]	** < 0.0001**	1.1 [0.72; 1.5]	** < 0.0001**
Women				
Never smoking (ref.)	–	–	–	–
> 0– < 10	0.22 [0.048; 0.40]	**0.012**	0.093 [-0.14; 0.33]	0.43
≥ 10– < 20	0.54 [0.34; 0.73]	** < 0.0001**	0.31 [0.0058; 0.62]	**0.046**
≥ 20– < 30	0.41 [0.18; 0.63]	**0.00037**	0.10 [-0.24; 0.45]	**0**.56
≥ 30	0.70 [0.47; 0.93]	** < 0.0001**	0.53 [0.17; 0.88]	**0.0038**
Estimates for augmentation index				
Men				
Never smoking (ref.)	–	–	–	–
> 0– < 10	3.3 [1.5; 5.2]	**0.00040**	4.4 [2.2; 6.5]	** < 0.0001**
≥ 10– < 20	9.1 [7.1; 11]	** < 0.0001**	10 [7.2; 13]	** < 0.0001**
≥ 20– < 30	12 [9.4; 14]	** < 0.0001**	14 [11; 17]	** < 0.0001**
≥ 30	14 [13; 16]	** < 0.0001**	17 [14; 20]	** < 0.0001**
Women				
Never smoking (ref.)	–	–	–	–
> 0– < 10	3.0 [0.98; 5.1]	**0.0039**	3.2 [0.55; 5.9]	**0.018**
≥ 10– < 20	8.9 [6.7; 11]	** < 0.0001**	6.1 [2.6; 9.5]	**0.00061**
≥ 20– < 30	10 [7.3; 13]	** < 0.0001**	11 [6.7; 15]	** < 0.0001**
≥ 30	10 [7.8; 13]	** < 0.0001**	12 [7.5; 16]	** < 0.0001**

Beta estimates and 95% confidence intervals are derived from a linear regression model modelling for arterial stiffness. Pack-years were modelled as categories (the reference category was never smoking)

Statistically significant *P* values (*P* < 0.05) are given in bold

**Model 1 was adjusted for age and augmentation index was additionally adjusted for height and heart rate. In women further adjustment for postmenopausal status, intake of oral contraceptives, and hormone replacement therapy was done

^#^Model 2 was additionally adjusted for arterial hypertension, waist-to-height ratio, diabetes mellitus, dyslipidemia, family history of myocardial infarction or stroke, socioeconomic status, alcohol consumption, physical activity, depression, smoking prior to the examination, prevalent cardiovascular disease (compromising congestive heart failure, coronary artery disease, myocardial infarction, stroke, atrial fibrillation, and peripheral artery disease), and medication use (diabetic drugs, antithrombotic agents, antihypertensives, diuretics, beta-blockers, calcium channel blocker, agents acting on the renin–angiotensin–aldosterone system, and lipid modifying agents)

**Table 5:** Sex-specific associations between pack-years of smoking in former smokers and markers of arterial stiffness

	Model 1** Beta estimate [95% CI]	*p* value	Model 2# Beta estimate [95% CI]	*p* value
Pack-years of smoking in former smokers	Estimates for stiffness index			
Men				
Never smoking (ref.)	–	–	–	–
> 0– < 5	0.26 [0.13; 0.39]	** < 0.0001**	0.22 [0.077; 0.36]	**0.0025**
≥ 5– < 10	0.46 [0.22; 0.71]	**0.00025**	0.47 [0.20; 0.75]	**0.00071**
≥ 10	0.56 [0.14; 0.97]	**0.0082**	0.52 [0.056; 0.99]	**0.028**
Women				
Never smoking (ref.)	–	–	–	–
> 0– < 5	0.71 [− 0.025; 0.17]	0.15	0.037 [− 0.070; 0.14]	0.50
≥ 5– < 10	0.038 [− 0.27; 0.35]	0.81	− 0.21 [− 0.57; 0.15]	0.26
≥ 10	1.3 [0.61; 2.0]	**0.00021**	1.3 [0.53; 2.1]	**0.0011**
	Estimates for augmentation index			
Men				
Never smoking (ref.)	–	–	–	–
> 0– < 5	2.9 [2.0; 3.7]	** < 0.0001**	2.4 [1.4; 3.4]	** < 0.0001**
≥ 5– < 10	6.2 [4.5; 7.9]	** < 0.0001**	5.6 [3.8; 7.5]	** < 0.0001**
≥ 10	7.6 [5.1; 10]	** < 0.0001**	5.9 [3.0; 8.8]	** < 0.0001**
Women				
Never smoking (ref.)	–	–	–	–
> 0– < 5	1.9 [0.74; 3.0]	**0.0011**	2.3 [1.0; 3.5]	**0.00033**
≥ 5– < 10	2.7 [− 0.62; 6.1]	0.11	5.4 [1.7; 9.1]	**0.0046**
≥ 10	− 6.5 [− 15; 2.1]	0.14	− 0.45 [− 11; 9.9]	0.93

Beta estimates and 95% confidence intervals are derived from a linear regression model modelling for arterial stiffness. Pack-years were modelled as categories (the reference category was never smoking)

Statistically significant *P* values (*P* < 0.05) are given in bold

**Model 1 was adjusted for age and augmentation index was additionally adjusted for height and heart rate. In women further adjustment for postmenopausal status, intake of oral contraceptives, and hormone replacement therapy was done

^#^Model 2 was additionally adjusted for arterial hypertension, waist-to-height ratio, diabetes mellitus, dyslipidemia, family history of myocardial infarction or stroke, socioeconomic status, alcohol consumption, physical activity, depression, prevalent cardiovascular disease (compromising congestive heart failure, coronary artery disease, myocardial infarction, stroke, atrial fibrillation, and peripheral artery disease), and medication use (diabetic drugs, antithrombotic agents, antihypertensives, diuretics, beta-blockers, calcium channel blocker, agents acting on the renin–angiotensin–aldosterone system, and lipid modifying agents)

**Table 6:** Sex-specific associations between heavy smoking and markers of arterial stiffness

	Model 1** Beta estimate [95% CI]	*P* value	Model 2# Beta estimate [95% CI]	*P* value
Pack-years of smoking in current smokers				
Men				
Never smoking (ref.)	–	–	–	–
< 20	0.55 [0.35; 0.75]	** < 0.0001**	0.45 [0.16; 0.75]	**0.0023**
≥ 20	1.1 [0.89; 1.3]	** < 0.0001**	0.97 [0.60; 1.3]	** < 0.0001**
Women				
Never smoking (ref.)	–	–	–	–
< 20	0.36 [0.23; 0.50]	** < 0.0001**	0.14 [− 0.083; 0.36]	0.22
≥ 20	0.54 [0.38; 0.71]	** < 0.0001**	0.23 [− 0.067; 0.53]	0.13
Estimates for augmentation index				
Men				
Never smoking (ref.)		–	–	–
< 20	5.9 [4.4; 7.3]	** < 0.0001**	5.6 [3.6; 7.7]	** < 0.0001**
≥ 20	14 [12; 15]	** < 0.0001**	14 [11; 17]	** < 0.0001**
Women				
Never smoking (ref.)		–	–	–
< 20	5.5 [3.9; 7.1]	** < 0.0001**	3.9 [1.4; 6.4]	**0.0021**
≥ 20	10 [8.3; 12]	** < 0.0001**	10 [7.0; 14]	** < 0.0001**

Beta estimates and 95% confidence intervals are derived from a linear regression model modelling for arterial stiffness. Pack-years were modelled as categories (the reference category was never smoking)

Statistically significant *P* values (*P* < 0.05) are given in bold

**Model 1 was adjusted for age and augmentation index was additionally adjusted for height and heart rate. In women further adjustment for postmenopausal status, intake of oral contraceptives, and hormone replacement therapy was done

^#^Model 2 was additionally adjusted for arterial hypertension, waist-to-height ratio, diabetes mellitus, dyslipidemia, family history of myocardial infarction or stroke, socioeconomic status, alcohol consumption, physical activity, depression, smoking prior to the examination, prevalent cardiovascular disease (compromising congestive heart failure, coronary artery disease, myocardial infarction, stroke, atrial fibrillation, and peripheral artery disease), and medication use (diabetic drugs, antithrombotic agents, antihypertensives, diuretics, beta-blockers, calcium channel blocker, agents acting on the renin–angiotensin–aldosterone system, and lipid modifying agents)

Augmentation index was independently and inversely associated with years since quitting smoking in former smokers and followed a dose-dependent relationship, whereas the independent and invers association with SI resulted in a rather non-linear weaker relationship in the total sample (Supplementary Table S5). This was also observed in the case of the sex-specific analyses, showing that SI was less linearly and weakly related to years since quitting smoking in both men and women with overall stronger effect estimates for men. In the case of AI, a dose-dependent association with years since quitting smoking was observed in men, whereas this association was less monotone and weaker in women (Table 7).

**Table 7:** Sex-specific associations between years since quitting smoking in former smokers and markers of arterial stiffness

	Model 1** Beta estimate [95% CI]	*p* value	Model 2# Beta estimate [95% CI]	*p* value
Years since quitting smoking in former smokers	Estimates for stiffness index			
Men				
Current smoking (ref.)	–	–	–	–
> 0– < 5	− 0.25 [− 0.52; 0.019]	0.068	− 0.16 [− 0.50; 0.19]	0.38
≥ 5– < 10	− 0.40 [− 0.65; − 0.14]	**0.0021**	− 0.18 [− 0.51; 0.16]	0.30
≥ 10– < 20	− 0.64 [− 0.85; − 0.42]	** < 0.0001**	− 0.47 [− 0.77; − 0.18]	**0.0018**
≥ 20– < 30	− 0.67 [− 0.90; − 0.45]	** < 0.0001**	− 0.45 [− 0.76; − 0.14]	**0.0040**
≥ 30	− 0.54 [− 0.76; − 0.31]	** < 0.0001**	− 0.33 [− 0.64; − 0.016]	**0.040**
Women				
Current smoking (ref.)	–	–	–	–
> 0– < 5	− 0.36 [− 0.60; − 0.12]	**0.0035**	− 0.16 [− 0.48; 0.15]	0.31
≥ 5– < 10	− 0.27 [− 0.48; − 0.054]	**0.014**	− 0.048 [− 0.34; 0.24]	0.75
≥ 10– < 20	− 0.38 [− 0.56; − 0.19]	** < 0.0001**	− 0.0052 [− 0.27; 0.26]	0.97
≥ 20– < 30	− 0.26 [− 0.45; − 0.075]	**0.0063**	0.030 [− 0.23; 0.30]	0.82
≥ 30	− 0.41 [− 0.62; − 0.20]	**0.00014**	− 0.17 [− 0.45; 0.12]	0.25
Estimates for augmentation index				
Men				
Current smoking (ref.)	–	–	–	–
> 0– < 5	− 3.1 [− 5.1; − 1.1]	**0.0020**	− 1.7 [− 4.2; 0.82]	0.19
≥ 5– < 10	− 5.4 [− 7.2; − 3.5]	** < 0.0001**	− 3.3 [− 5.7; − 0.90]	**0.0071**
≥ 10– < 20	− 6.5 [− 8.0; − 4.9]	** < 0.0001**	− 4.8 [− 7.0; − 2.7]	** < 0.0001**
≥ 20– < 30	− 8.3 [− 10; − 6.7]	** < 0.0001**	− 6.2 [− 8.4; − 3.9]	** < 0.0001**
≥ 30	− 8.7 [− 10; − 7.0]	** < 0.0001**	− 6.0 [− 8.3; − 3.8]	** < 0.0001**
Women				
Current smoking (ref.)	–	–	–	–
> 0– < 5	− 4.8 [− 7.7; − 1.9]	**0.0013**	− 0.033 [− 3.7; 3.6]	0.99
≥ 5– < 10	− 5.5 [− 8.1; − 2.9]	** < 0.0001**	− 2.4 [− 5.8; 0.95]	0.16
≥ 10– < 20	− 4.9 [− 7.1; − 2.7]	** < 0.0001**	− 1.2 [− 4.2; 1.9]	0.45
≥ 20– < 30	− 6.5 [− 8.7; − 4.2]	** < 0.0001**	− 3.4 [− 6.4; − 0.32]	**0.031**
≥ 30	− 4.1 [− 6.6; − 1.6]	** < 0.0013**	− 1.8 [− 5.0; − 0.60]	0.29

Beta estimates and 95% confidence intervals are derived from a linear regression model modelling for arterial stiffness. Years since quitting were modelled as categories (the reference category was current smoking)

Statistically significant *P* values (*P* < 0.05) are given in bold

**Model 1 was adjusted for age and augmentation index was additionally adjusted for height and heart rate. In women further adjustment for postmenopausal status, intake of oral contraceptives, and hormone replacement therapy was done

^#^Model 2 was additionally adjusted for arterial hypertension, waist-to-height ratio, diabetes mellitus, dyslipidemia, family history of myocardial infarction or stroke, socioeconomic status, alcohol consumption, physical activity, depression, smoking prior to the examination, prevalent cardiovascular disease (compromising congestive heart failure, coronary artery disease, myocardial infarction, stroke, atrial fibrillation, and peripheral artery disease), and medication use (diabetic drugs, antithrombotic agents, antihypertensives, diuretics, beta-blockers, calcium channel blocker, agents acting on the renin–angiotensin–aldosterone system, and lipid modifying agents)

## Discussion

In the present study, sex-specific associations between chronic current as well as former cigarette smoking and arterial stiffness quantified by SI and AI were investigated in a large population-based cohort. Regardless of sex, smoking was dose-dependently associated with increased arterial stiffness and wave reflection. Fortunately, an increasing number of years since quitting smoking in former smokers was associated with an improvement in arterial compliance in both sexes. The portion of current smokers and consumed pack-years were higher in men than in women in the present study. In line, women overall revealed a more beneficial cardiovascular risk profile compared to men and were associated with less cardiovascular comorbidities. Arterial stiffness (SI) was higher in men than in women and both AI and SI had a higher correlation to current smoking in men than in women in linear regression. With raising pack-years a higher correlation to arterial stiffness was revealed in both sexes, whereas this effect was stronger in men. In men and women heavy smoking correlated with increased arterial stiffness, but male sex was associated with higher values of AI and SI in heavy smokers compared to the female sex. In line with these findings, the regression of arterial stiffness after quitting smoking was present in both sexes, but AI and SI declined stronger in men than in women. Of interest, prospective analyses revealed an association between current as well as former smoking and SI in men but not in women after further adjustment in linear regression models. AI, in contrast, was associated with current smoking but not with former smoking in both sexes. Altogether, the present study indicates that smoking may constitute an independent risk factor for increased arterial stiffness and wave reflection in both men and women.

To our knowledge, this is the first assessment of the sex-specific association between current and former cigarette smoking and arterial stiffness measured by SI and AI in a large population-based cohort. In accordance with the literature [22, 23], in the present study the prevalence of smoking was higher in men than in women. Until now, only a little amount of literature exists on possible sex differences regarding arterial stiffness due to smoking. In many available studies, the explanatory power is largely hampered by small numbers of study participants. In line with the results of the present study, an investigation of 147 smokers by Mozos et al. revealed a higher cumulative smoking exposure (pack-years) and an unbeneficial cardiovascular risk profile in men compared to women. The authors also found that an elevation of arterial stiffness required less exposure to cigarette smoke in women compared to men [24], which goes along with the results of the present study. Besides these findings, arterial stiffness increase was associated with smoking intensity (pack-years) in the overall sample, but no further sex differences regarding arterial stiffness and smoking were detected. Contrary to the findings of Mozos et al. [24], in the present study heavy smokers revealed higher values of arterial stiffness in both sexes, but in men a worse effect on SI and AI was seen compared to women, the results of the present study revealed lower arterial stiffening in women compared to men. In a Japanese study on more than 6,500 healthy subjects, in whom AI was measured, wave reflection was higher in women than in men, although the interaction of blood pressure and smoking with arterial stiffness was observed only in men. The authors concluded that smoking/hypertension interaction might be more detrimental regarding arterial stiffness in men compared to women [25]. In contrast to AI, aortic stiffness was shown to be higher in female smokers than in male smokers [24, 26], indicating varying stiffness patterns of the vascular system in men and women due to smoking exposure [13]. In the present study, smoking affected arterial stiffness in men more intense than in women, whereas conversely smoking cessation had a more beneficial effect on arterial stiffness in males than in females. Until now, the effect of smoking cessation on arterial stiffness is barely investigated. While most existing studies revealed an improvement of arterial stiffness after quitting smoking [27–29] and this positive effect was even seen with nicotine replacement therapy [30], a study from the Netherlands found no decrease in arterial wall thickness and stiffness after two years of smoking cessation [31]. However, this and most of the previous studies only comprised small study samples. In contrast, the present study represents the largest study sample so far, investigating the effect of smoking cessation on arterial stiffness in a sex-specific manner. In the present study, a dose-dependent association between smoking and arterial stiffness was demonstrated and smoking cessation resulted in an improvement in arterial elasticity in both sexes. Scarce prospective data exist on the effect of smoking on arterial stiffness. Tomiyama et al. found a dose-dependent correlation between cigarette smoking and accelerated arterial stiffness in Japanese adults within a five to six years follow-up [32]. These results are in line with our findings. Beyond, in the present study—to our knowledge for the first time—the sex-specific prospective effect of smoking on arterial stiffness was assessed: current and former smoking were associated with higher SI in men but not in women, whereas AI correlated with current smoking but not with former smoking in both sexes.

Arterial stiffness represents a hallmark of aging and is related to premature vascular aging. It is induced by several cardiometabolic disorders like diabetes mellitus and arterial hypertension. By this, several differences in clinical impact between men and women were demonstrated [13]. While the incidence of CVD is relatively low in premenopausal women, the risk to develop CVD increases considerably after menopause [33]. In the literature, increasing evidence demonstrates an independent association between arterial stiffness cardiovascular disease and mortality, whereas this association was almost doubled in women compared to men [7, 8, 13]. In this context, women suffer more often from coronary microvascular dysfunction and heart failure with preserved ejection fraction, both representing conditions which are associated with arterial stiffness and drivers of CVD mortality [13, 34–37]. Also, sex-specific differences in cognitive function due to arterial stiffening were shown [38] and female sex as well as smoking were identified as independent predictors of human vascular smooth muscle cell stiffening [39]. Regarding specific arterial regions, carotid intima-media thickness was found to be increased in smoking men but not in women [40] and acute exposure to passive smoking influenced the arterial pressure waveform in males but not in females [41]. As one main reason for sex-specific differences in arterial stiffness, sex hormones and vascular receptors are in the focus of research efforts. Especially estrogen was identified to be protective against CVD in premenopausal women baring a lower CVD risk compared to men of the same age. However, postmenopausal women develop CVD approximately ten years later than men, what is widely explained by estrogen loss after menopause [13, 42, 43]. Even an alternating arterial stiffness within the menstrual cycle is discussed in regard to the fluctuating levels of estrogen [13, 44, 45]. The use of hormone-based oral contraception is assumed to promote arterial stiffness and CVD due to interference of sex hormone levels [46]. However, the age- and hormone-mediated impact on arterial stiffness is hard to distinguish since age might outmatch possible hormone effects. Nevertheless, an influence of sex hormones on arterial stiffness cannot be excluded and future investigations are required to elucidate hormone-based effects on vessel arterial elasticity [47].

Pathophysiologically, smoking might promote arterial stiffness by affecting multiple mechanisms such as alteration of the lipid and glucose metabolism, induction of inflammation and oxidative stress or impairment of endothelial function [4, 28]. Lipid metabolism is influenced by smoking due to altered catecholamine release and lipoprotein lipase activity, which lead to elevated triglyceride and low-density lipoprotein blood levels as well as reduction of high-density lipoproteins [48]. By this, atherosclerosis is advanced and hence presumably arterial stiffness [28, 49]. Smoking induces insulin resistance [50] and increases the risk for diabetes mellitus [51]. The risk for developing diabetes mellitus was shown to remain high in heavy smokers even after smoking cessation [52]. Both, insulin resistance and diabetes mellitus cause arterial stiffening [28, 53], which has a considerable clinical impact since arterial stiffness was identified as an independent risk factor for mortality in people with diabetes mellitus [54]. Interestingly, recent investigations likewise revealed vice versa arterial stiffness as a risk factor for the development of diabetes mellitus [55, 56]. One of the main underlying mechanisms of arterial stiffness in a diabetic state is represented by nitric oxide dysregulation [53]. In this context, oxidative stress itself is also caused by smoking [57] and leads to arterial stiffness [58] by the production of reactive oxygen species, which leads to a decrease of nitric oxide by inhibition of nitric oxide synthase [59]. Additionally, smoking causes a decrease in endogenous antioxidant levels with consecutive reduced protection from oxidative stress [60]. A procoagulatory state is provoked by an imbalance of the intraplatelet redox state due to reduced bioactivity of platelet-derived nitric oxide [28, 61] and current smoking does not only influence the arterial vascular system, but also the venous system with increased risk for venous thromboembolism [12]. In the context of impaired synthesis of nitric oxide, also endothelial function has shown to be reduced as a consequence of smoking advancing arterial stiffness [4, 28, 62]. Furthermore, smoking induces inflammation by a mismatch of pro- and anti-inflammatory cytokine expression. Inflammation is strongly associated with arterial stiffness and additionally provokes arterial calcification and remodeling [28, 63–65]. Arterial stiffness was shown to be higher in people with arterial hypertension, and the risk for arterial hypertension is elevated by smoking. Furthermore, it has been shown that smoking weakens the stiffness-lowering effect of antihypertensive medication. It is supposed that smoking and arterial hypertension affect arterial stiffness by similar mechanisms, mainly by promoting oxidative stress [4, 28, 66–68].

### Strengths and limitations

The present study has several limitations, which should be addressed here. Firstly, the overall generalizability of present findings is mostly limited to middle-aged subjects of European ancestry. Furthermore, again due to the study design no detailed examination of smoking behavior within the GHS was possible and a self‐reported assessment of smoking status might be susceptible to information bias. It is known that studies based on self-report are at risk to underestimate smoking status [69], which also cannot be excluded in the GHS. Also, the standard definition of never smoking used in our and other studies can be seen as critical as it defines never-smokers as the group of non-daily and non-regular smokers and thus not truly reflects never smoking.

This study also has several strengths: The GHS is the largest study to date, assessing the sex‐specific relation of smoking status to arterial stiffness and wave reflection. Another strength consists in the simultaneous assessment of two methods of arterial compliance, which are complementary to each other, thereby providing a more comprehensive evaluation of smoking effects on the vasculature. Taken together, the present study compared the influence of arterial stiffness and wave reflection in men and women in a large and highly standardized population-based European cohort. Differences between men and women could be shown, whereas in both sexes smoking had a vast impact on arterial stiffness and wave reflection.

## Supplementary Information

Below is the link to the electronic supplementary material.

Supplementary file1 (DOCX 248 KB)
