# Supplementary material for: Chronic cigarette smoking is associated with increased arterial stiffness in men and women: evidence from a large population-based cohort
Source: Clin Res Cardiol. 2022 Sep 6;112(2):270–84. doi: 10.1007/s00392-022-02092-1 (PMC9898409; doi:10.1007/s00392-022-02092-1)
Supplement: Supplementary file 1 — Supplementary file1 (DOCX 248 KB) [file 392_2022_2092_MOESM1_ESM.docx]

***Supplemental material***

**Chronic cigarette smoking is associated with increased arterial stiffness in men and women: evidence from a large population-based cohort**

Omar Hahad, PhD^1,2*^; Volker H. Schmitt, MD^1,2*^; Natalie Arnold, MD^1,2,3^; Karsten Keller, MD^1,4,5^; Jürgen H. Prochaska, MD^2,4,6^; Philipp S. Wild, MD, MSc ^2,4,6^; Andreas Schulz, PhD^6^; Karl J. Lackner, MD^2,7^; Norbert Pfeiffer, MD^8^; Irene Schmidtmann, PhD^9^; Matthias Michal, MD^10^; Jörn M. Schattenberg, MD^11^; Oliver Tüscher, MD^12^; Andreas Daiber, PhD^1,2^; Thomas Münzel, MD^1,2^

**Running title*:*** *Hahad et al. – Smoking and arterial stiffness*

^1^ Department of Cardiology – Cardiology I, University Medical Center of the Johannes Gutenberg-University Mainz, Mainz, Germany

^2^ German Center for Cardiovascular Research (DZHK), partner site Rhine-Main, Mainz, Germany

^3^ Department of Cardiology, University Heart and Vascular Center Hamburg, Hamburg, Germany

^4^ Center for Thrombosis and Hemostasis, University Medical Center of the Johannes Gutenberg-University Mainz, Mainz, Germany

^5^ Medical Clinic VII, Department of Sports Medicine, University Hospital Heidelberg, Heidelberg, Germany

^6^ Preventive Cardiology and Preventive Medicine, Department of Cardiology, University Medical Center of the Johannes Gutenberg-University Mainz, Mainz, Germany

^7^ Institute of Clinical Chemistry and Laboratory Medicine, University Medical Center of the Johannes Gutenberg-University Mainz, Mainz, Germany

^8^ Department of Ophthalmology, University Medical Center of the Johannes Gutenberg-University Mainz, Mainz, Germany

^9^ Institute of Medical Biostatistics, Epidemiology & Informatics, University Medical Center of the Johannes Gutenberg-University Mainz, Mainz, Germany

^10^ Department of Psychosomatic Medicine and Psychotherapy, University Medical Center of the Johannes Gutenberg-University Mainz, Mainz, Germany

^11^ Metabolic Liver Research Program, I. Department of Medicine, University Medical Center of the Johannes Gutenberg-University Mainz, Mainz, Germany

^12^ Department of Psychiatry and Psychotherapy, University Medical Center of the Johannes Gutenberg-University Mainz, Mainz, Germany

* Omar Hahad and Volker H. Schmitt contributed equally and share first authorship

**Table S1. Characteristics of the study sample by smoking status (*N*=14,975).***

|  | **Never**  **(*N*=6,863)** | **Former**  **(*N*=5,201)** | **Current**  **(*N*=2,911)** |
| --- | --- | --- | --- |
| **Characteristic** |  |  |  |
| Female sex – no. (%) | 3,960 (57.7) | 2,116 (40.7) | 1,335 (45.9) |
| Age – years | 55.3±11.8 | 56.7±10.4 | 51.3±9.7 |
| Physical activity** | 7.29±3.79 | 7.15±3.98 | 7.92±4.41 |
| Heart rate – beats per minute | 69.2±10.8 | 68.6±11.1 | 69.2±10.6 |
| Height – cm | 169±10 | 172±9 | 171±9 |
| Waist-to-height ratio# | 0.55±0.08 | 0.57±0.08 | 0.55±0.08 |
| Postmenopausal status – no. (%) (among women) | 2,692 (68.1) | 1,499 (70.9) | 814 (61.0) |
| Hormone replacement therapy – no. (%) (among women) | 425 (10.8) | 255 (12.1) | 123 (9.3) |
| Intake of oral contraceptives – no. (%) (among women) | 248 (6.3) | 110 (5.2) | 90 (6.8) |
| Alcohol consumption above tolerable limit – no. (%)‡ | 1,209 (17.6) | 1,415 (27.2) | 740 (25.4) |
| Socioeconomic status† | 13.06±4.64 | 13.02±4.37 | 12.25±4.22 |
| Depression – no. (%)§ | 466 (6.9) | 352 (6.9) | 314 (11.0) |
| Smoking | | | |
| Pack-years | - | 1.57 (0.57/3.54) | 18.70 (8.40/31.79) |
| Years since quitting | - | 18.0 (8.00/29.00) | - |
| Passive smoking – no. (%) | 934 (13.6) | 952 (18.3) | - |
| Smoked < 12 hours prior to examination – no. (%) | - | - | 2,114 (72.6) |
| Traditional cardiovascular risk factors – no. (%) | | | |
| Arterial hypertension | 3,424 (49.9) | 2,878 (55.4) | 1,147 (39.4) |
| Diabetes mellitus | 558 (8.2) | 601 (11.6) | 228 (7.8) |
| Dyslipidemia | 2,136 (31.2) | 2,019 (38.9) | 1,009 (34.7) |
| Family history of myocardial infarction or stroke | 1,435 (20.9) | 1,211 (23.3) | 670 (23.0) |
| Cardiovascular comorbidities – no. (%) | | | |
| Congestive heart failure | 476 (6.9) | 430 (8.3) | 242 (8.3) |
| Coronary artery disease | 217 (3.2) | 329 (6.4) | 94 (3.3) |
| Myocardial infarction | 123 (1.8) | 234 (4.5) | 85 (2.9) |
| Stroke | 104 (1.5) | 128 (2.5) | 47 (1.6) |
| Atrial fibrillation | 1,169 (17.0) | 1,003 (19.3) | 527 (18.1) |
| Peripheral artery disease | 189 (2.8) | 207 (4.0) | 106 (3.7) |
| Measurements of arterial stiffness | | | |
| Stiffness index – m/s | 7.23±2.08 | 7.81±2.28 | 7.81±2.29 |
| Augmentation index – % | 15.93±20.30 | 17.59±19.66 | 20.55±22.00 |
| Medication – no. (%)§§ | | | |
| Diabetic drugs (A10) | 382 (5.6) | 406 (7.9) | 130 (4.5) |
| Antithrombotic agents (B01) | 738 (10.9) | 827 (16.0) | 274 (9.5) |
| Antihypertensives (C02) | 74 (1.1) | 63 (1.2) | 18 (0.6) |
| Diuretics (C03) | 359 (5.3) | 318 (6.2) | 109 (3.8) |
| Beta-blockers (C07) | 1,148 (16.9) | 1,011 (19.6) | 370 (12.9) |
| Calcium channel blocker (C08) | 490 (7.2) | 437 (8.5) | 158 (5.5) |
| Agents acting on the renin-angiotensin-aldosterone system (C09) | 1,545 (22.8) | 1,478 (28.7) | 503 (17.5) |
| Lipid modifying agents (C10) | 843 (12.4) | 877 (17.0) | 258 (9.0) |

* Plus-minus values are means ± standard deviation and two values in parentheses are medians with 25^th^ and 75^th^ percentiles.

** Physical activity score was calculated by multiplying total minutes of activity by the intensity score displayed per 1000-units with higher values indicating higher physical activity.

# Waist-to-height ratio is the waist circumference divided by the body height in centimeters.

‡ Alcohol consumption above tolerable limit denotes >24 g per day for men and >12 g per day for women.

† Socioeconomic status score ranges from 3 to 21 with higher values indicating higher status.

§ Caseness of depression was indicated by a PHQ-9 score ≥10.

§§ Medication is labelled with the anatomical therapeutic chemical-code.

**Table S2. Associations between smoking status and markers of arterial stiffness.***

|  | **Model 1****  **Beta estimate [95% CI]** | ***P* value** | **Model 2#**  **Beta estimate [95% CI]** | ***P* value** |
| --- | --- | --- | --- | --- |
| **Estimates for stiffness index** | | | | |
| Never smoking (ref.) | - | - | - | - |
| Current smoking | 0.66 [0.57; 0.75] | **<0.0001** | 0.39 [0.22; 0.56] | **<0.0001** |
| Former smoking | 0.22 [0.14; 0.30] | **<0.0001** | 0.22 [0.13; 0.31] | **<0.0001** |
| **Estimates for augmentation index** | | | | |
| Never smoking (ref.) | - | - | - | - |
| Current smoking | 8.7 [7.9; 9.5] | **<0.0001** | 6.3 [4.7; 7.8] | **<0.0001** |
| Former smoking | 2.8 [2.2; 3.5] | **<0.0001** | 3.2 [2.5; 4.0] | **<0.0001** |

* Beta estimates and 95% confidence intervals are derived from a linear regression model modelling for arterial stiffness. Current and former smoking were compared to never smoking (reference category).

** Model 1 was adjusted for sex and age and augmentation index was additionally adjusted for height and heart rate.

# Model 2 was additionally adjusted for arterial hypertension, waist-to-height ratio, diabetes mellitus, dyslipidemia, family history of myocardial infarction or stroke, socioeconomic status, alcohol consumption, physical activity, depression, passive smoking, smoking prior to examination, prevalent cardiovascular disease (compromising congestive heart failure, coronary artery disease, myocardial infarction, stroke, atrial fibrillation, and peripheral artery disease), and medication use (diabetic drugs, antithrombotic agents, antihypertensives, diuretics, beta-blockers, calcium channel blocker, agents acting on the renin-angiotensin-aldosterone system, and lipid modifying agents).

**Table S3. Associations between pack-years of smoking in current smokers and markers of arterial stiffness.***

|  | **Model 1** Beta estimate [95% CI]** | ***P* value** | **Model 2#** **Beta estimate [95% CI]** | ***P* value** |
| --- | --- | --- | --- | --- |
| **Pack-years of smoking in current smokers** | **Estimates for stiffness index** | | | |
| Never smoking (ref.) | - | - | - | - |
| >0–<10 | 0.32 [0.17; 0.47] | **<0.0001** | 0.23 [0.038; 0.42] | **0.0019** |
| ≥10–<20 | 0.65 [0.49; 0.82] | **<0.0001** | 0.60 [0.34; 0.85] | **<0.0001** |
| ≥20–<30 | 0.67 [0.49; 0.85] | **<0.0001** | 0.64 [0.36; 0.91] | **<0.0001** |
| ≥30 | 1.0 [0.87; 1. 2] | **<0.0001** | 0.90 [0.63; 1. 2] | **<0.0001** |
|  | **Estimates for augmentation index** | | | |
| Never smoking (ref.) | - | - | - | - |
| >0–<10 | 3.4 [2.0; 4.8] | **<0.0001** | 4.1 [2.4; 5.8] | **<0.0001** |
| ≥10–<20 | 9.2 [7.6; 11] | **<0.0001** | 8.1 [5.8; 10] | **<0.0001** |
| ≥20–<30 | 11 [9.1; 13] | **<0.0001** | 12 [9.9; 15] | **<0.0001** |
| ≥30 | 14 [12; 15] | **<0.0001** | 15 [13; 17] | **<0.0001** |

* Beta estimates and 95% confidence intervals are derived from a linear regression model modelling for arterial stiffness. Pack-years were modelled as categories (the reference category was never smoking).

** Model 1 was adjusted for sex and age and augmentation index was additionally adjusted for height and heart rate.

# Model 2 was additionally adjusted for arterial hypertension, waist-to-height ratio, diabetes mellitus, dyslipidemia, family history of myocardial infarction or stroke, socioeconomic status, alcohol consumption, physical activity, depression, smoking prior to examination, prevalent cardiovascular disease (compromising congestive heart failure, coronary artery disease, myocardial infarction, stroke, atrial fibrillation, and peripheral artery disease), and medication use (diabetic drugs, antithrombotic agents, antihypertensives, diuretics, beta-blockers, calcium channel blocker, agents acting on the renin-angiotensin-aldosterone system, and lipid modifying agents).

**Table S4. Associations between heavy smoking and markers of arterial stiffness.***

|  | **Model 1** Beta estimate [95% CI]** | ***P* value** | **Model 2#** **Beta estimate [95% CI]** | ***P* value** |
| --- | --- | --- | --- | --- |
| **Pack-years of smoking in current smokers** | **Estimates for stiffness index** | | | |
| Never smoking (ref.) | - | - | - | - |
| <20 | 0.46 [0.34; 0.58] | **<0.0001** | 0.32 [0.13; 0.50] | **0.00076** |
| ≥20 | 0.87 [0.74; 1.0] | **<0.0001** | 0.66 [0.42; 0.91] | **<0.0001** |
|  | **Estimates for augmentation index** | | | |
| Never smoking (ref.) | - | - | - | - |
| <20 | 5.9 [4.8; 7.0] | **<0.0001** | 5.0 [3.4; 6.6] | **<0.0001** |
| ≥20 | 12 [11; 14] | **<0.0001** | 13 [10; 15] | **<0.0001** |

* Beta estimates and 95% confidence intervals are derived from a linear regression model modelling for arterial stiffness. Pack-years were modelled as categories (the reference category was never smoking).

** Model 1 was adjusted for sex and age and augmentation index was additionally adjusted for height and heart rate.

# Model 2 was additionally adjusted for arterial hypertension, waist-to-height ratio, diabetes mellitus, dyslipidemia, family history of myocardial infarction or stroke, socioeconomic status, alcohol consumption, physical activity, depression, smoking prior to examination, prevalent cardiovascular disease (compromising congestive heart failure, coronary artery disease, myocardial infarction, stroke, atrial fibrillation, and peripheral artery disease), and medication use (diabetic drugs, antithrombotic agents, antihypertensives, diuretics, beta-blockers, calcium channel blocker, agents acting on the renin-angiotensin-aldosterone system, and lipid modifying agents).

**Table S5. Associations between years since quitting smoking in former smokers and markers of arterial stiffness.***

|  | **Model 1** Beta estimate [95% CI]** | ***P* value** | **Model 2#** **Beta estimate [95% CI]** | ***P* value** |
| --- | --- | --- | --- | --- |
| **Years since quitting smoking in former smokers** | **Estimates for stiffness index** | | | |
| Current smoking (ref.) | - | - | - | - |
| >0–<5 | -0.29 [-0.48; -0.10] | **0.0024** | -0.12 [-0.37; 0.12] | 0.32 |
| ≥5–<10 | -0.34 [-0.52; -0.17] | **<0.0001** | -0.12 [-0.35; 0.10] | 0.29 |
| ≥10–<20 | -0.53 [-0.67; -0.38] | **<0.0001** | -0.25 [-0.46; -0.049] | **0.015** |
| ≥20–<30 | -0.49 [-0.64; -0.34] | **<0.0001** | -0.22 [-0.43; -0.00095] | **0.041** |
| ≥30 | -0.47 [-0.63; -0.31] | **<0.0001** | -0.21 [-0.43; 0.012] | 0.064 |
|  | **Estimates for augmentation index** | | | |
| Current smoking (ref.) | - | - | - | - |
| >0–<5 | -3.7 [-5.4; -2.0] | **<0.0001** | -0.91 [-3.0; 1.1] | 0.40 |
| ≥5–<10 | -5.4 [-7.0; -3.9] | **<0.0001** | -1.8 [-4.8; -0.83] | **0.0055** |
| ≥10–<20 | -5.8 [-7.1; -4.5] | **<0.0001** | -3.1 [-4.9; -1.3] | **0.00067** |
| ≥20–<30 | -7.4 [-8.8; -6.0] | **<0.0001** | -4.8 [-6.7; -3.0] | **<0.0001** |
| ≥30 | -6.7 [-8.2; -5.3] | **<0.0001** | -4.0 [-5.9; -2.2] | **<0.0001** |

* Beta estimates and 95% confidence intervals are derived from a linear regression model modelling for arterial stiffness. Years since quitting were modelled as categories (the reference category was current smoking).

** Model 1 was adjusted for sex and age and augmentation index was additionally adjusted for height and heart rate.

# Model 2 was additionally adjusted for arterial hypertension, waist-to-height ratio, diabetes mellitus, dyslipidemia, family history of myocardial infarction or stroke, socioeconomic status, alcohol consumption, physical activity, depression, smoking prior to examination, prevalent cardiovascular disease (compromising congestive heart failure, coronary artery disease, myocardial infarction, stroke, atrial fibrillation, and peripheral artery disease), and medication use (diabetic drugs, antithrombotic agents, antihypertensives, diuretics, beta-blockers, calcium channel blocker, agents acting on the renin-angiotensin-aldosterone system, and lipid modifying agents).

**Table S6. Associations between pack-years of smoking in former smokers and markers of arterial stiffness.***

|  | **Model 1** Beta estimate [95% CI]** | ***P* value** | **Model 2#** **Beta estimate [95% CI]** | ***P* value** |
| --- | --- | --- | --- | --- |
| **Pack-years of smoking in former smokers** | **Estimates for stiffness index** | | | |
| Never smoking (ref.) | - | - | - | - |
| >0–<5 | 0.20 [0.12; 0.28] | **<0.0001** | 0.17 [0.083; 0.26] | **0.00016** |
| ≥5–<10 | 0.40 [0.21; 0.59] | **<0.0001** | 0.40 [0.19; 0.61] | **0.00018** |
| ≥10 | 0.69 [0.37; 1.0] | **<0.0001** | 0.75 [0.38; 1.1] | **<0.0001** |
|  | **Estimates for augmentation index** | | | |
| Never smoking (ref.) | - | - | - | - |
| >0–<5 | 2.6 [1.9; 3.4] | **<0.0001** | 2.9 [2.1; 3.6] | **<0.0001** |
| ≥5–<10 | 5.7 [4.1; 7.3] | **<0.0001** | 6.6 [4.9; 8.3] | **<0.0001** |
| ≥10 | 6.7 [4.0; 9.4] | **<0.0001** | 7.3 [4.3; 10] | **<0.0001** |

* Beta estimates and 95% confidence intervals are derived from a linear regression model modelling for arterial stiffness. Pack-years were modelled as categories (the reference category was never smoking).

** Model 1 was adjusted for sex and age and augmentation index was additionally adjusted for height and heart rate.

# Model 2 was additionally adjusted for arterial hypertension, waist-to-height ratio, diabetes mellitus, dyslipidemia, family history of myocardial infarction or stroke, socioeconomic status, alcohol consumption, physical activity, depression, prevalent cardiovascular disease (compromising congestive heart failure, coronary artery disease, myocardial infarction, stroke, atrial fibrillation, and peripheral artery disease), and medication use (diabetic drugs, antithrombotic agents, antihypertensives, diuretics, beta-blockers, calcium channel blocker, agents acting on the renin-angiotensin-aldosterone system, and lipid modifying agents).

**A. Stiffness index B. Augmentation index**

**Figure S1. Effect plots demonstrating the relationship between smoking status and markers of arterial stiffness.** Adjusted mean values are derived from a linear regression model and beta estimates, 95% confidence intervals, and adjustment are shown in Table S2.

**A. Stiffness index B. Augmentation index**

**Figure S2. Effect plots demonstrating the relationship between pack-years of smoking in current smokers and markers of arterial stiffness.** Adjusted mean values are derived from a linear regression model and beta estimates, 95% confidence intervals, and adjustment are shown in Table S3.
